# Frequency of Deleterious Germline Variants in HER2-Low Breast Cancer Patients Using a Hereditary Multipanel Gene Testing

**DOI:** 10.3390/cimb46080471

**Published:** 2024-07-25

**Authors:** Janaina Pontes Batista Cassoli, Ítalo Fernandes, Leonardo Carvalho, Milena Fernandes, Ana Fernanda Centrone, Letícia Taniwaki, Rita de Cássia Lima, Uelson Donizeti Rocioli Junior, Igor Wanderley Reis Dias, Patrícia Taranto, Juliana Beal, Fernanda Teresa de Lima, Fernando Moura, Miguel Cendoroglo, Sergio Eduardo Alonso Araújo, Pedro Luiz Serrano Uson Junior

**Affiliations:** 1 Center for Personalized Medicine, Hospital Israelita Albert Einstein, São Paulo 05652000, Brazil; janaina.batista@einstein.br (J.P.B.C.); leonardo.carvalho@einstein.br (L.C.); milena.fernandes@einstein.br (M.F.); leticia.taniwaki@einstein.br (L.T.); rita.lima@einstein.br (R.d.C.L.); uelson.junior@einstein.br (U.D.R.J.); igor.dias@einstein.br (I.W.R.D.); patricia.taranto@einstein.br (P.T.); bealjuliana@gmail.com (J.B.); fernanda.lima@einstein.br (F.T.d.L.); f.moura@einstein.br (F.M.); miguel.cendoroglo@einstein.br (M.C.); sergio.araujo@einstein.br (S.E.A.A.); 2 Department of Hematology and Oncology, Hospital Israelita Albert Einstein, São Paulo 05652000, Brazil; italo0206@gmail.com (Í.F.); ana.centrone@einstein.br (A.F.C.)

**Keywords:** breast cancer, HER2-Low, genetics, germline genetic testing, *BRCA*

## Abstract

HER2-Low is defined as low levels of HER2 expression, based on a score of 1+ on immunohistochemical (IHC) assay or as an IHC score of 2+ and negative results on in situ hybridization (ISH or FISH). They are a heterogeneous population of breast cancers that vary in prognosis and sensitivity to systemic treatments. The frequency and clinical characteristics of pathogenic germline variants (PGVs) in HER2-Low breast cancer (BC) patients is not defined. We analyzed results from patients with BC who underwent multi-gene panel testing (MGPT) (maximum 145 genes) between 2018–2019. We reclassified HER-2 status accordingly. Relationships between the variables of interest were assessed by adopting the proportional regression Cox models. Of a total of 167 BC patients who underwent MGPT, half were hormone-receptor-positive. The median age was 45 years. About two thirds of the patients were in the earlier stage of BC. A total of 57% of the cases were reclassified as HER-2-negative or -Low. PGVs were found in 19% of the patients overall, as follows: seven *BRCA1*, four *BRCA2*, two *ATM*, one *ATR*, two *CFTR*, three *CHEK2*, one *FANCA*, one *MERTK*, one *MLH1*, three *MUTYH*, one *RAD50*, three *RAD51C*, one *RECQL4*, and two *TP53* mutations. In HER2-Low, 26.5% of the patients had PGVs, and in the overall cohort, this was 19.8%. In conclusion, differences in the prevalence of deleterious germline mutations in HER2-Low BC patients compared to non-HER2-Low BC patients were identified. Similar alterations in *BRCA* were observed in this group of patients compared to the overall cohort. Germline genetic tests should be evaluated in larger cohorts of patients with HER2-Low status to better address the findings.

## 1. Introduction

Breast cancer (BC) is the most common cancer worldwide [1]. According to GLOBOCAN 2020, 2,261,419 new cases of the disease were estimated in 2020, which represents 11.7% of all cancers [1]. BC was responsible for 6.9% of cancer deaths in 2020 and was the leading cause of cancer death in women [1]. There are many risk factors associated with the development of breast cancer: female gender; increasing age; being white; obesity in peri- and post menopause; alcohol and tobacco use; exposure to therapeutic ionizing radiation; benign breast diseases and dense breasts; hormonal factors (endogenous estrogen, menopausal hormone therapy, contraceptives); reproductive factors (earlier menarche or later menopause, nulliparity, increasing age at first full-term pregnancy); personal and family history of breast cancer; and genetic mutations [2,3,4,5,6,7,8,9,10,11,12,13]. It is well known that hereditary mutations increase the risk of BC [14]. It is estimated that about 10% of the cases are associated with pathogenic germline variants (PGVs) [14].

When these genetic mutations are present, the absolute risk of breast cancer greatly increases, reaching up to 60% in some cases [15]. The risk depends on the penetrance (proportion of those with a specific genotype that display the phenotype) of the gene. The high-penetrance breast cancer genes are *TP53*; *BRCA1* and *BRCA2* (increasing the risk by more than 60%); *PALB2* (increasing the risk by 41 to 60%); *PTEN* (increasing the risk by 40 to 60%); C*DH1* (increasing the risk by 41 to 60%); and *STK11* (increasing the risk by 32% to 54%). The moderate-penetrance breast cancer genes are *ATM*, *CHEK2*, *NF1, BARD1*, *RAD51C*, and *RAD51D* (increasing the risk by 20 to 40%) [15].

Breast cancer is a heterogeneous disease, with different molecular subtypes that have biological distinctness and different behaviors, mostly defined based on gene expression profiling. Subtypes of BC are classified by the expression of tumor markers: estrogen receptor (ER), progesterone receptor (PR), and HER2 status [16,17,18,19]. HR^+^/HER2^−^ is associated with the Luminal subtype that accounts for about 70% of patients; HER2^+^ (HER2-enriched) accounts for about 15–20%; and HR^−^/HER2^−^ (triple-negative) accounts for about 15% of cases [17,18,19,20].

Triple-negative breast cancer is more likely to recur than the other two subtypes, with 85% 5-year breast cancer-specific survival for stage I triple-negative tumors vs. 94% to 99% for hormone-receptor-positive and ERBB2-positive types [19]. Systemic therapy for nonmetastatic breast cancer is determined by subtype: patients with hormone-receptor-positive tumors receive endocrine therapy, and a minority receive chemotherapy as well; patients with ERBB2-positive tumors receive ERBB2-targeted antibody or small-molecule inhibitor therapy combined with chemotherapy; and patients with triple-negative tumors receive chemotherapy [19]. Local therapy for all patients with nonmetastatic breast cancer consists of surgical resection, with consideration of postoperative radiation if lumpectomy is performed. Increasingly, some systemic therapy is delivered before surgery. Tailoring postoperative treatment based on preoperative treatment response is under investigation. Metastatic breast cancer is treated according to subtype, with goals of prolonging life and palliating symptoms [19]. 

International guidelines generally recommend germline testing in patients diagnosed with breast cancer aged 50 years and younger regardless of molecular subtype or family history [15]. The BRIDGES study included more than 80,000 patients to characterize tumors associated with BC susceptibility genes in a large scale. They found that there was substantial heterogeneity in the distribution of intrinsic subtypes by PGV [20]. *RAD51C*, *RAD51D*, and *BARD1* variants were associated mainly with triple-negative disease (OR, 6.19 [95%CI, 3.17–12.12]; OR, 6.19 [95%CI, 2.99–12.79]; and OR, 10.05 [95%CI, 5.27–19.19], respectively). *CHEK2* variants were associated with all subtypes (with ORs ranging from 2.21 to 3.17), except for triple-negative disease. For *ATM* variants, the association was strongest for the hormone receptor (HR)+ERBB2− high-grade subtype (OR, 4.99; 95%CI, 3.68–6.76). *BRCA1* was associated with an increased risk of all subtypes, but the ORs varied widely, being highest for triple-negative disease (OR, 55.32; 95%CI, 40.51–75.55). *BRCA2* and *PALB2* variants were also associated with triple-negative disease. *TP53* variants were most strongly associated with HR+ERBB2+ and HR–ERBB2+ subtypes. Tumors occurring in pathogenic variant carriers were of higher grade. For most genes and subtypes, a decline in ORs was observed with increasing age. Together, the nine genes were associated with 27.3% of all triple-negative tumors in women 40 years or younger [20].

The identification of these variants is important for genetic counseling. Risk-reducing bilateral radical mastectomy can be discussed in the presence of alterations in genes of moderate to high penetrance. Furthermore, current treatments are available that target the pathogenic mutation. As an example, the OlympiA trial showed that among high-risk patients, early HER2-negative breast cancer, and germline *BRCA1* or *BRCA2* pathogenic or likely pathogenic variants, adjuvant olaparib after the completion of local treatment and neoadjuvant or adjuvant chemotherapy was associated with significantly longer survival free of invasive or distant disease than was placebo [21]. 

The amplification of the HER-2 gene causes the upregulation of key signaling pathways that control cell growth and survival. In breast cancer patients, HER-2 overexpression correlates with an aggressive phenotype and poor prognosis. HER-2, therefore, has become the focus of many anti-cancer therapeutic approaches. Trastuzumab (Herceptin), a humanized monoclonal antibody directed against the extracellular domain of HER-2, was the first FDA-approved HER-2-targeted therapy for the treatment of metastatic breast cancer [22,23,24]. After the approval of trastuzumab, several studies were designed, with new drugs in different scenarios with important gains in results [25,26,27]. 

In patients with HER2-positive metastatic breast cancer, the CLEOPATRA study showed that the addition of pertuzumab to trastuzumab and docetaxel, as compared with the addition of placebo, significantly improved the median overall survival to 56.5 months and extended the results of previous analyses showing the efficacy of this drug combination [27]. However, there are some BCs without HER-2 amplification, overexpression, or both, with a large proportion of these patients expressing low levels of HER2, defined as a score of 1+ on immunohistochemical (IHC) analysis or as an IHC score of 2+ and negative results on in situ hybridization (ISH or FISH). These tumors constitute a heterogeneous population including both hormone-receptor-positive and hormone-receptor-negative breast cancers that vary in prognosis and sensitivity to systemic treatments [28,29]. For these groups of patients, trastuzumab–deruxtecan (formerly DS-8201), an antibody–drug conjugate consisting of a humanized anti-HER2 monoclonal antibody linked to a topoisomerase I inhibitor payload through a tetrapeptide-based cleavable linker, has been demonstrated to be highly effective [30].

Although data have been gathered, the PGV prevalence in this group of patients is not yet clear. Considering the multiple options of systemic treatment discussed briefly, the molecular status of HER2-Low needs to be identified. In this study, we aim to describe the frequency and clinical characteristics of deleterious germline variants in HER2-Low BC patients tested with germline multigene panel testing (MGPT) and compare it with overall patients.

## 2. Materials and Methods

We retrospectively analyzed the results from a prospective database of 167 patients with BC who underwent commercial germline multigene panel testing (MGPT) (that included the analysis of 20 to 145 genes) with an analysis of point variant and copy number variation (CNV), treated at Hospital Israelita Albert Einstein in Sao Paulo, Brazil, between 2018 and 2019. This project was approved by the local IRB with project number CAAE: 81744017.6.0000.0071. We compared the findings of patients with HER2-Low BC with overall BC population evaluated in the period. The groups were compared accordingly the HER2 reclassification and the number of pathogenic germline variants detected by MGPT. Relationships between the variables of interest were assessed by adopting the proportional regression Cox models.

Formalin-fixed, paraffin-embedded tissues with a standard thickness of 4–5 µm were evaluated by breast pathologists. We assessed HER2 status, estrogen receptor (ER), progesterone receptor (PR), and Ki-67 by IHC. Tumors with HER2 IHC 2+ were analyzed by FISH and determined as positive or negative according to ASCO/CAP guidelines. Tumors with HER2 0 by IHC were considered HER2-negative; HER2 1+ or 2+ by IHC with FISH-negative were considered HER2-Low; and those with HER2 3+ or HER2 2+ by IHC and FISH-positive were considered HER2-positive. Tumors were considered HR-positive if more than 1% of invasive cells were ER/PR-positive according to IHC assay. HER2 IHC was assessed using Polyclonal Rabbit Anti-Human c-erB-2 Oncoprotein from Agilent Technologies Singapore (International) Ple Ltd., Singapore.

## 3. Results and Discussion

In this study, we describe the frequency and clinical characteristics of deleterious germline variants in HER2-Low BC patients tested with germline multigene panel testing (MGPT) and compare it with overall cohort. We found a higher incidence of heterozygous PGV in the HER2-Low group when compared to the HER2-positive or -negative groups. 

**Figure 1:**
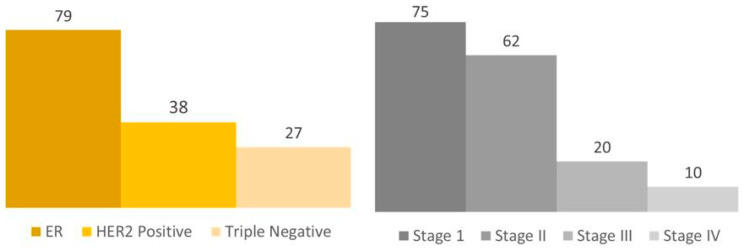
Overall cohort. Legend: The number of patients is exemplified in each column.

The database included 167 BC female patients in the center who underwent MGPT. The median age was 45.8 years. There were 75 (45%) stage I, 62 (37.1%) stage II, 20 (11.9%) stage III, and 10 (6%) stage IV patients. From this group, data about the BC subtype were available in 144 cases. There were 79 (47.3%) hormone receptor (HR)-positive, 38 (22.7%) HER2-positive, and 27 (16%) triple-negative breast cancer (TN) patients [Figure 1]. 

A total of 134 cases were reclassified as HER2-negative, HER2-positive, or HER2-Low [Table 1]. In the 34 patients with HER2-Low BC, 27 (80%) patients were estrogen-receptor-positive and 7 (20%) were negative, and 26 (76%) were progesterone-receptor-positive and 8 (24%) were negative. The median age for the HER2-Low group was 45 years, and there were 19 (56%) stage I, 12 (35%) stage II, 3 (9%) stage III, and no stage IV patients. In the 62 patients with HER2-negative BC, 54 (87%) patients were estrogen-receptor-positive and 8 (13%) cases were negative, and 50 (80%) patients were progesterone-receptor-positive and 12 (20%) were negative. The median age for the HER2-negative group was 44 years, and there were 36 (58%) stage I, 20 (32%) stage II, 5 (9%) stage III, and one stage IV patients. In the 38 patients with HER2-positive BC, 28 (73%) patients were estrogen-receptor-positive and 10 (27%) were negative, and 23 (60%) cases were progesterone-receptor-positive and 15 (40%) were negative. The median age for the HER2-positive group was 44 years, and there were 18 (47%) stage I, 9 (23%) stage II, 4 (10%) stage III, and 7 (20%) stage IV patients [Table 1].

In our study, the median age is about 45 years old. It is an age that is recommended by international guidelines for germline testing [15]. Out of the total group, 75 patients (45%) were classified as stage I, 62 patients (37.1%) as stage II, 20 patients (11.9%) as stage III, and 10 patients (6%) as stage IV. The higher incidence of patients in earlier stages of the disease is probably due to private care services, also reflecting the greater access of the overall cohort to health care systems. 

Much research has studied whether the presence of deleterious germline variants influences the molecular subtype of breast cancer and whether it is associated with tumor heterogeneity and worse prognosis. Data have shown that PGV in high penetrance genes including BRCA1 was associated with all subtypes, with higher incidence in triple-negative BC (OR, 55.32; 95%CI, 40.51–75.55) [21]. BRCA2 and PALB2 variants were also associated with triple-negative disease. TP53 variants were most strongly associated with HR+HER2+ and HR–HER2+ subtypes. In moderate penetrance genes, CHEK2 variants were associated with all subtypes (with ORs ranging from 2.21 to 3.17), except for triple-negative disease [21]. For ATM variants, the association was strongest for the hormone receptor HR+HER2− high-grade BC subtype (OR, 4.99; 95%CI, 3.68–6.76) [21], but the prevalence in HER2-Low was not described.

Heterozygous pathogenic or likely pathogenic variants (PGVs or LPGVs) were found in 33 patients overall, as follows: seven *BRCA1*, four *BRCA2*, two *ATM*, one *ATR*, two *CFTR*, three *CHEK2*, one *FANCA*, one *MERTK*, one *MLH1*, three *MUTYH*, one *RAD50*, three *RAD51C*, one *RECQL4*, and two *TP53* mutations [Table 2]. 

**Figure 2:**
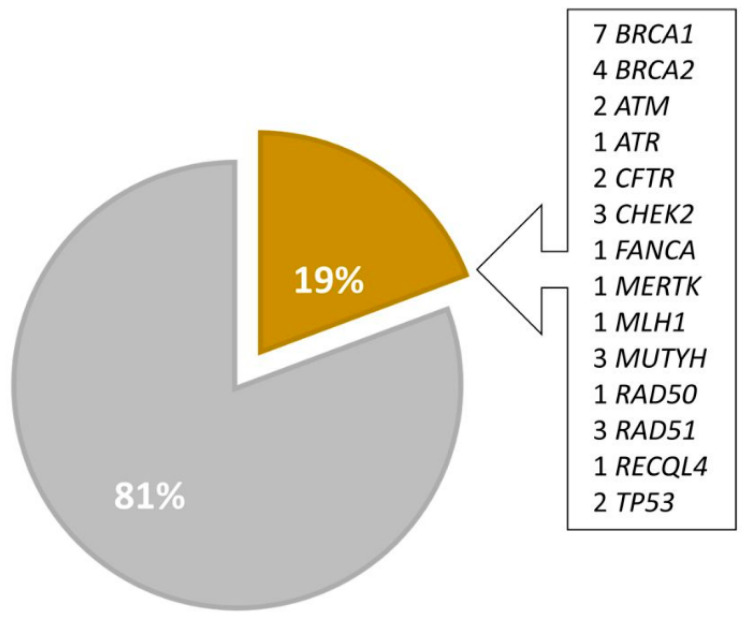
Frequency of mutations in overall population. Legend: Percentage of cases with pathogenic germline variants highlighted in yellow.

Interestingly, in HER2-Low patients, a PGV or LPGV was identified in 9 of 34 patients, which represents 26.5% of the patients, as follows: one *BRCA1*, one *BRCA2*, one *TP53*, one *RAD51C*, one *RAD50*, one *CHEK2*, one *MERTK*, and two *MUTYH* variants; meanwhile the incidence in the overall cohort was 19.8% [Figure 2]. Furthermore, most of these variants were identified in genes that increase the risk of BC by 40% to 60% [15]. However, some findings may be incidental and not associated with a higher risk. Genes with low penetrance for BC were also identified, for example, a heterozygous variant of the MUTYH gene for which, so far, we do not have sufficient data to confirm an association with an increased risk of BC [15]. Some are common variants, and their potential carcinogenic effect is associated with biallelic MUTYH variants, which are, in turn, associated with the development of gastrointestinal polyposis [18]. In addition, access to multicancer panels could result in incidental findings of pathogenic or likely pathogenic variants that are not associated with the patient’s clinical condition, as seen in some of the patients.

Additionally, the incidence of deleterious variants both in tumors with a low expression of HER2 (26.5%) and in the cohort in general (19.8%) surpassed the literature data estimate of around 10% [14]. It is suggested that these results are due to specialized medical assistance in the treatment of BC in a private tertiary hospital, which includes universal germline genetic testing, since the finding of a pathogenic variant impacts the treatment, prevention, and care of relatives of patients with established syndromes [31].

HER2-Low patients were not statistically related to a higher or lower incidence of deleterious variants (*p* = 0.17). No statistical differences were identified in the prevalence of mutations in *BRCA1* or *BRCA2* or homologous recombinant repair genes (*p* = 0.9). Furthermore, no statistical significance was observed in the clinical characteristics such as age, histological type, tumor grade, or ki67 in patients with HER2-Low compared to the overall cohort. Other PGVs identified in patients with HER2 unknown status can be found in Table 3.

**Figure 3:**
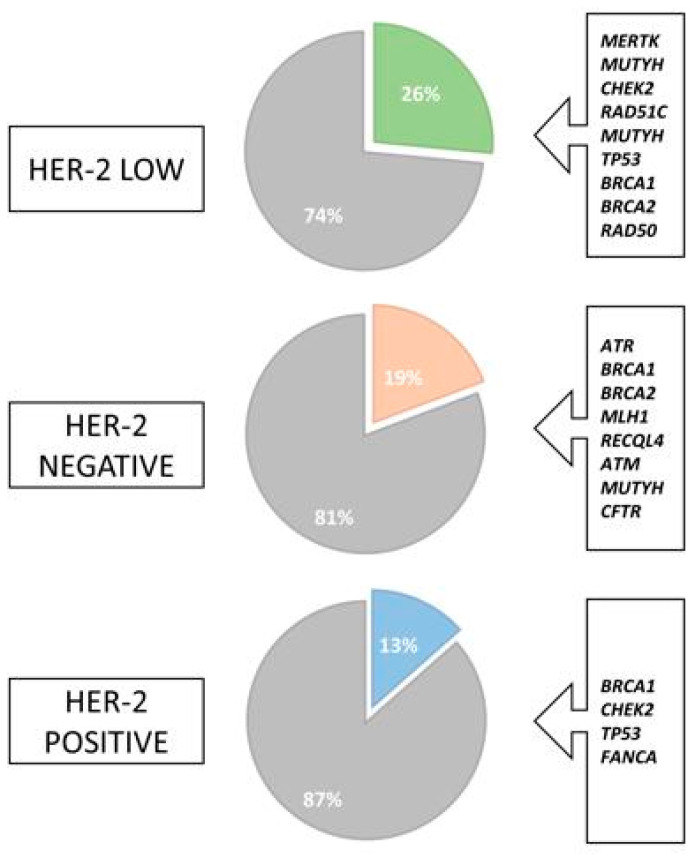
Frequency of deleterious mutations between HER2 subtypes of breast cancer. Legend: Green, orange, and blue colors highlight the percentage of cases with pathogenic germline variants in each group according to HER2 expression.

Although not statistically significant, a higher number of PGVs were identified in the HER2-Low group (26%) when compared to the HER2-positive (13%) and HER2-negative cases (19%) [Figure 3]. We were unable to establish a statistically significant association between the HER2-Low and the pathogenic variant, a finding that may be related to the small sample size. As demonstrated, HER2-Low is a heterogeneous disease, and the association between germline variants and the molecular subtype is still unclear.

## 4. Conclusions

In conclusion, this study analyzed a database of 167 breast cancer patients who underwent molecular genetic germline testing. The results showed the distribution of patients among different stages and breast cancer molecular subtypes. Additionally, the presence of pathogenic or likely pathogenic variants was investigated, with interesting findings in the HER2-Low group. HER2-Low BC patients seem to have a different pattern of incidence of germline genetic findings compared to the overall breast cancer population, although the number of patients was relatively small. Further research is needed to better understand the implications of deleterious germline variants in the molecular subtype and their impact on disease management.

## Figures and Tables

**Table 1:** Main characteristics of HER2-tested patients after reclassification.

		HER2-Low (n = 34)	HER2-Negative (n = 62)	HER2-Positive (n = 38)
Age, y (median)		45	44	44
Female		34	62	38
Histology	In situ	1	2	2
	Ductal	28	47	31
	Lobular	3	7	3
	Other	2	6	2
Pathological Stage	ypT0	2	2	7
	pT1	21	39	15
	ypT1	1	2	3
	pT2	8	9	3
	ypT2	0	4	1
	pT3	1	0	0
	ypT3	0	1	1
	pT4	0	0	0
	NS	1	4	8
Lymph Nodes	pN0	20	35	12
	ypN0	2	6	9
	pN1	7	13	4
	ypN1	1	1	2
	pN2	3	2	2
	ypN2	0	2	1
	pN3	0	0	0
	NI	1	3	8
Histologic Grade	1	4	13	2
	2	18	32	17
	3	11	11	17
	NS	1	6	2
Estrogen Receptor	Positive	27	54	28
	Negative	7	8	10
Progesterone Receptor	Positive	26	50	23
	Negative	8	12	15
HER2 IHQ	0	0	62	0
	1	21	0	0
	2	13	0	4
	3	0	0	34
Ki-67	<20	11	36	3
	≥20	22	25	35
	NS	1	1	0

Legend: NS: No specified.

**Table 2:** Descriptions of pathogenic and likely pathogenic mutations between HER2 subtypes.

Gene	Transcript	Variant
HER2-Low		
*MERTK*	NM_006343	c.2785_2786dup;p.(Ile930ThrfsTer3)
*MUTYH*	NM_001128425.2	c.289C>T;p.(Arg97*)
*CHEK2*	Unavailable	Unavailable
*RAD51C*	NM_058216	c.656T>C;p.(Leu219Ser)
*MUTYH*	Not reported	c.1145G>A
*TP53*	NM_000546	c.1010G>A;p.(Arg337His)
*BRCA1*	NM_007294	c.212+1del;p?
*BRCA2*	NM_000059	c.7868A>G;p.(His2623Arg)
*RAD50*	NM_005732	c.1875C>G;p.(Tyr625*)
HER2-Negative		
*ATR*	NM_001184	c.1652T>A;p.(Leu551Ter)
*BRCA1*	NM_007294	c.211A>G;p.(Arg71Gly)
*BRCA1*	NM_007294	c.190T>C;p.(Cys63Arg)
*BRCA2*	NM_000059	c.2808_2811del;p.(Ala938Profs*21)
*MLH1*	NM_000249	c.394G>C;p.(D132H)
*RECQL4*	NM_004260	c.2464-1G>C (Splice Aceptor)
*ATM*	NM_000051	c.8395_8404del;p.(Phe2799LysfsTer4)
*BRCA1*	NM_007294	c.5266dypC;p.(Gln1756Profs*74)
*CFTR*	NM_000492	c.1521_1523del;p.(Phe508del)
*MUTYH*	NM_001128425	c.1187G>A;p.(Gly396Asp)
*ATM*	Unavailable	Unavailable
*CFTR*	Unavailable	c.1210-34TG[12]T[5] (intronic)
HER2-Positive		
*BRCA1*	NM_007294	c.4414delC;p.(Leu1472Phefs*33)
*CHEK2*	NM_007194	c.349A>G;p.(Arg117Gly)
*CHEK2*	NM_007194	c.470T>C;p.(Ile157Thr)
*TP53*	NM_000546	c.1010G>A;p.(Arg337His)
*FANCA*	NM_000135	c.718C>T;p.(Gln140Ter)

Legend: fs: frameshift; *: stop codon.

**Table 3:** Descriptions of pathogenic and likely pathogenic mutations among patients with unknown HER2 status.

Gene	Transcript	Variant and Protein
*BRCA1*	Unavailable	Unavailable
*CHEK2*	NM_007194	c.349A>G;p.(Arg117Gly)
*RAD51C*	NM_058216	c.404G>A;p.(Cys135Yhr)
*BRCA1*	NM_007294	c.192T>G;p.(Cys64Trp)
*BRCA2*	NM_000059	c.6202dup;p.(Ile2068Asnfs*10)
*BRCA2*	NM_000059	c.5216dup;p.(Tyr1739*)
*RAD51C*	NM_058216	c.404G>A;p.(Cys135Tyr)

Legend: *: stop codon.

## Data Availability

All data related to this work are under the protection of Hospital Israelita Albert Einstein and under LGPT. The data can be made available upon reasonable request.
